# Tarlov Cysts and Premature Ejaculation

**DOI:** 10.1007/s10508-024-02815-7

**Published:** 2024-02-16

**Authors:** Yuanyuan Liu, Dalin Sun, Zhenghong Gao, Zhongjiang Wang, Baofang Jin

**Affiliations:** 1 Southeast University School of Medicine, Nanjing, China; 2 Andrology Department of Integrative Medicine, Zhongda Hospital, School of Medicine, Southeast University, Nanjing, 210009 China; 3 People’s Hospital of Jingjiang, Taizhou, China; 4 Department of Radiology, Zhongda Hospital, School of Medicine, Southeast University, Nangjing, China

**Keywords:** Sacral cyst, Tarlov cyst, Premature ejaculation, Pelvic Pain

## Abstract

Tarlov cysts adjacent to the spinal cord are usually asymptomatic and found incidentally via magnetic resonance imaging. On rare occasions, they increase in size to produce symptoms resembling disk herniation. We report a rare case of a sacral cyst resulting in premature ejaculation in a 32-year-old man who presented with pelvic pain and acquired premature ejaculation. Spinal nerve root decompression, excision of intraspinal Tarlov cyst, and spinal nerve root adhesion release surgery significantly improved his pain and premature ejaculation at a six-month follow-up.

## Introduction

### Physiology of Mammalian Ejaculation Types

Ejaculation is divided into two types of emission and expulsion (Alwaal et al., 2015). Emission is the first phase of ejaculation and solely dependent on contractions of the smooth muscles of the prostate, seminal vesicles, and vas deferens, and its initiation can be voluntarily controlled before reaches the posterior urethra. All organs participating in the emission phase receive dense autonomic innervations composed of sympathetic (T12–L1) and parasympathetic axons (S1–S3). The second phase of ejaculation is expulsion that is a passage of seminal fluid from the posterior urethra to the external urethral meatus depending on the spinal cord reflex, banking on contractions of the pelvic floor muscles in addition to the bulbospongiosus and ischiocavernosus muscles (El-Hamd et al., 2019). Therefore, abnormal nerve conduction may be a potential risk factor for premature ejaculation (PE). PE is a frequent male sexual complaint or sexual disturbance found in andrology with the prevalence rate reaching up 20–30% (Wisard & Audette, 2008). However, lumbosacral disease such as Tarlov cysts with acquired PE is rarely reported.

### Tarlov Cysts

Tarlov cysts their presence may result from inflammation, trauma, congenital origin, and degenerative processes (Murphy et al., 2024). In clinical practice, the treatment of Tarlov cyst is performed by neurosurgeon, but it is also recommended with urology department that the presence of Tarlov cyst can be detected by MRI. The focus of its treatment is to relieve symptoms such as pain. If there are serious symptoms and the corresponding treatment cannot be relieved, the Talov cyst will be treated surgically. The cysts in the sacral canal compress the cauda equine nerve root, and the patient might show inflammatory characteristics associated with cauda equina syndrome (Greenhalgh et al., 2018). Some studies on cauda equina syndrome have shown that sacral cysts are associated with sexual dysfunction, but symptoms of PE have not been clearly shown. Mummaneni et al. reported that one of the eight patients with sacral cysts suffered from impotence (Mummaneni et al., 2000). Voyadzis et al. (2001) reported that two patients have sexual dysfunction of the ten patients with sacral cysts. Five of the twelve males reported by Galarza et al. (2021) have sexual dysfunction. Singh et al. (2009) reported a case of infertility caused by retrograde ejaculation caused by Talov's cyst, and the retrograde ejaculation was improved after operation. In the case described here, a Tarlov cyst was diagnosed as the cause of PE. The cyst was surgically removed from the patient to improve symptoms such as PE.

## Case Report

**Fig. 1:**
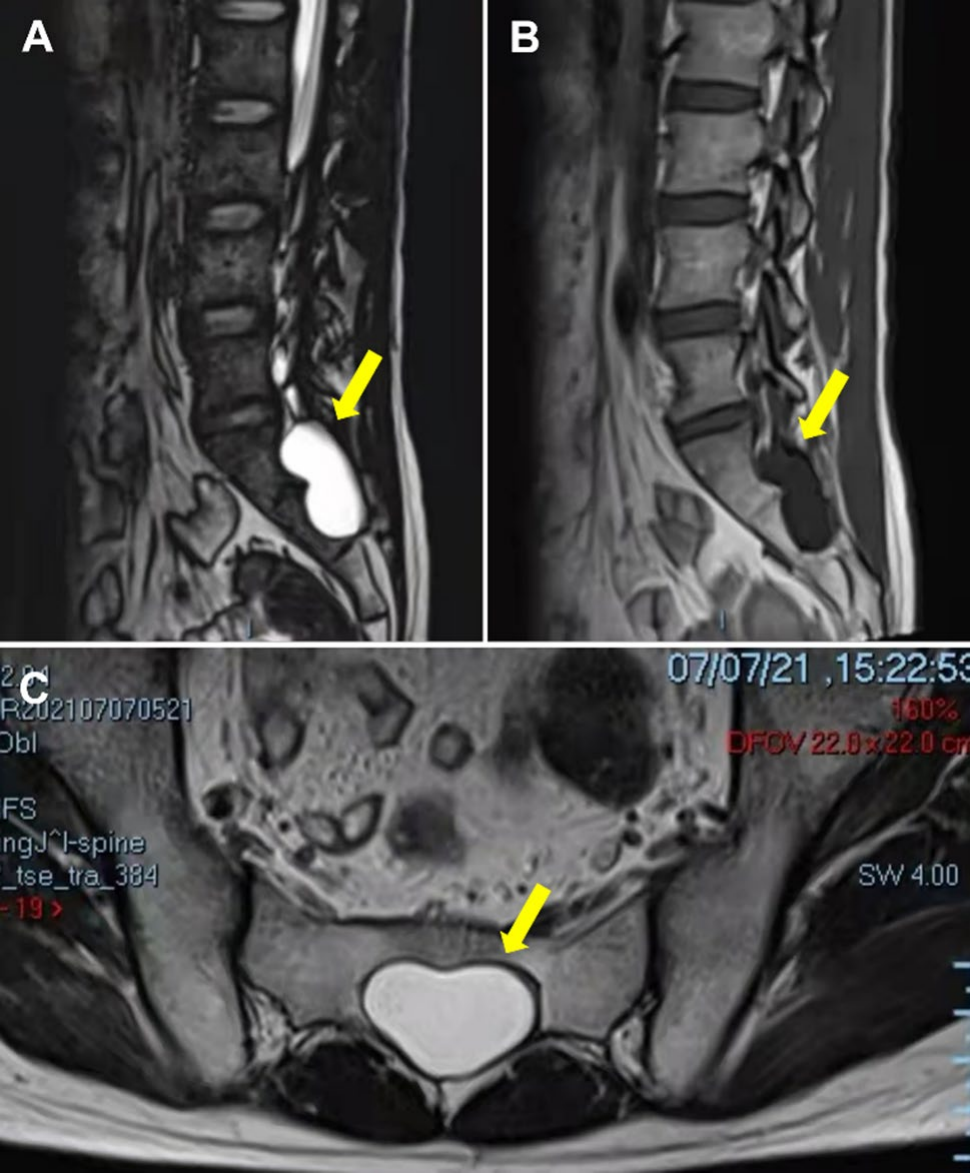
**A** A sagittal magnetic resonance (MR) scan shows abnormal spinal cancel signals at the T1W1 level. **B** A sagittal MR scan shows abnormal spinal cancel signals at the T2W1 level. **C** An axial MR scan shows abnormal spinal cancel signals at the T1W1 level. The Tarlov cyst is shown by an arrow in **A**–**C**

A 32-year-old man who presented with a 2-year history of PE, which had started 1.5 years before and had gradually aggravated and accompanied by priapism, pelvis pain and painful ejaculation. After an MRI of the lumbar spine, one oval lesion T2 signal shadows were observed in the L5–S2 sacral canal and accompanied by an expansion of the sacral canal and a compression of the sacral nerve root (Fig. 1). Thus, he was diagnosed with Tarlov cyst and PE at the local hospital.

## Method

To assess the severity of PE through intravaginal ejaculation latency (IELT) and premature ejaculation diagnostic tool (PEDT) after its initial presentation. Chronic pelvic pain symptoms caused by cauda equine compression are detected using the internationally accepted National Institutes of Health-Chronic Prostatitis Symptom Index (NIH-CPSI) score (Franco et al., 2018). The patients IELT Severity Score was 1 min, PEDT score was 18 points and NIH-CPSI score was 36 points. The patient had obvious PE and severe pain around the pelvis symptoms. Therefore, prompt surgical treatment is necessary.

## Results

The boundary of the lesion was expanded by the resection area along the boundary of the lesion, separated the tumor from the nerve root and from the spinal cord, and performed total resection of the tumor at Xinhua Hospital affiliated to Shanghai Jiao Tong University School of Medicine. PE and pain around the pelvis were significantly relieved. The IELT of this patient improved to 5 min, the PEDT score reduced significantly to 5 points, and NIH-CPSI score was decreased to 3 points determined by a follow-up conducted after six months postoperative. These results suggested that resection of sacral lesions can significantly improve priapism, painful ejaculation, pelvic pain, and PE caused by abnormal lumbar and sacral.

## Discussion

In sexual health surveys, PE is reported as the most common issues in men. PE is ejaculation occurring within less than one minute of vaginal penetration (Chung et al., 2015; Salonia et al., 2021; Sun et al., 2021). The prevalence of PE accounts for 20–30% of sexual dysfunction (Raveendran et al., 2021). Currently, assessment of patients with PE relies mainly on the use of validated questionnaires. PEDT and the stopwatch measurement of IELT are often used in the clinic (Althof et al., 2010). The etiology of PE is mainly divided into psychological factors and biological factors. Psychological factors include psychological pressure, early experience, sexual conditioning, anxiety, and the skill and frequency of sexual activities. Biological factors include penile hypersensitivity, hyperexcitatory ejaculatory reflex, hyperexcitability, endocrine disease, genetic susceptibility, and 5-HT receptor dysfunction (neurobiological theory), and chronic prostatitis and other urinary system diseases (El-Hamd et al., 2019). Additionally, neurological diseases, including lumbar disk herniation and spinal stenosis, have been proposed as causes of PE (Jin, 2015; Yazici et al., 2013). Lumbar disk herniation, spinal stenosis, and Tavlov cysts rarely show positive results in X-ray reports but can be demonstrated by the information of lesion information in MRI (Hornung et al., 2023; Sun et al., 2021).

### Conclusion

Most caudal cysts are asymptomatic, but some grow large enough to compress nearby nerves and cause some symptoms, such as lumbago, sciatica and leg pain. However, when patients come to urology or andrology with PE as the main manifestation, nerve symptoms and effective image-assisted examination are often ignored during diagnosis and treatment. In our opinion, spine factors and appropriate auxiliary examinations should be considered after excluding other pathogenic factors in an otherwise healthy male who has recently developed abnormal sexual function as shown in this case. Surgical resection may benefit PE patients with progressive symptoms of caudal cysts.

## Data Availability

The original materials in this report are available from the corresponding author upon reasonable request.
